# Reinforcement Learning-Based Adaptive Streaming Scheme with Edge Computing Assistance

**DOI:** 10.3390/s22062171

**Published:** 2022-03-10

**Authors:** Minsu Kim, Kwangsue Chung

**Affiliations:** Department of Electronics and Communications Engineering, Kwangwoon University, Seoul 01897, Korea; mskim@cclab.kw.ac.kr

**Keywords:** Dynamic Adaptive Streaming over HTTP (DASH), Quality of Experience (QoE), mobile edge computing (MEC), reinforcement learning

## Abstract

Dynamic Adaptive Streaming over HTTP (DASH) is a promising scheme for improving the Quality of Experience (QoE) of users in video streaming. However, the existing schemes do not perform coordination among clients and depend on fixed heuristics. In this paper, we propose an adaptive streaming scheme with reinforcement learning in edge computing environments. The proposed scheme improves the overall QoE of clients and QoE fairness among clients based on a state-of-the-art reinforcement learning algorithm. Edge computing assistance plays a role in providing client-side observations to the mobile edge, making agents utilize this information when generating a policy for multi-client adaptive streaming. We evaluated the proposed scheme through simulation-based experiments under various network conditions. The experimental results show that the proposed scheme achieves better performance than the existing schemes.

## 1. Introduction

According to the Cisco Annual Internet Report (2018–2023), the total number of global mobile subscribers will increase from 66% of the population in 2018 to 71% of the population by 2023 [1]. Video services such as Netflix and YouTube contribute to the major portion of Internet traffic. Since these services deliver videos over the Internet, providing a high Quality of Experience (QoE) to users is an important challenge in terms of network and service [2]. Dynamic Adaptive Streaming over HTTP (DASH) was standardized in 2011 as a solution for efficient and smooth video streaming. Using the existing HTTP infrastructure, DASH adapts the bitrate of video segments delivered over the network to improve resource utilization and the QoE for users [3,4,5]. Moreover, DASH has high scalability owing to the client-driven scheme that does not impose any modification to the HTTP server. Various studies based on DASH have been conducted over several years [6,7,8,9,10]. These schemes perform bitrate adaptation by determining the video bitrate fit to the measured available bandwidth, current buffer level, or other predicted conditions.

QoE refers to the degree of delight or annoyance of a user for an application or service. Many factors associated with network, device, user expectation, and content affect the QoE. In adaptive streaming, the QoE is determined by considering startup delay, packet loss rates, average video quality, video re-buffering events, and quality variations, etc. According to studies for the QoE, a user wants to view a video as clearly and smoothly as possible. The long startup delay prevents a user from viewing a video smoothly after the streaming session starts. The packet loss rate and average video quality are related to the clarity of play-back scenes to be watched by users. Once the video re-buffering events occur, the QoE is significantly degraded compared with the other influencing factors. The quality variations leading to abrupt changes of perceived quality also have negative effect on the QoE. Various schemes have been proposed to measure and evaluate the QoE, focusing on modeling using a combination of influencing factors. A linear combination QoE model is commonly used to measure the QoE in adaptive streaming [11]. We utilize this model for the proposed scheme. The QoE model based on linear combination is simple and makes it easy to represent how user experiences are shaped by the influencing factors. By considering subjective user experiences, the modeling method for cost-effective and real-time QoE measurements has been proposed [12]. Through subjective tests, this method models a correlation between the QoE and the Quality of Service (QoS) parameters, such as packet loss rate, delay, and requested video bitrate.

In general, many users stream videos through the same network. This causes the users to compete for the limited bandwidth of the network [13]. The greedy policy of the DASH-based bitrate adaptation degrades the QoE of users due to a lack of coordination among clients [14,15]. This problem leads to inefficient resource utilization and QoE unfairness among clients. Moreover, the existing schemes based on heuristics are not effective in optimizing the QoE of users in the environments where available bandwidth changes dynamically. The adaptive streaming scheme should address the following challenges to optimize the QoE of users. First, the overall QoE of clients should be improved by considering multi-client competition. Second, the intelligent bitrate adaptation should be performed to robustly adapt the video bitrate, even if network conditions change significantly. We utilize edge computing and reinforcement learning to improve the performance of multi-client adaptive streaming. The video bitrate is adjusted by using global knowledge for clients with the help of mobile edge computing. Through reinforcement learning, a bitrate adaptation policy is generated by mining information regarding the actual performance of past choices.

In this paper, we propose an adaptive streaming scheme with reinforcement learning in edge computing environments. The main contributions of the proposed scheme are as follows.

- We present a reinforcement learning-based mobile edge framework for multi-client adaptive streaming. The mobile edge collects the information of clients and feeds them as inputs into a neural network model to determine the bitrate of video segments to be requested.
- We formulate the multi-client QoE fairness problem as a Markov Decision Process (MDP) to define the optimization objectives of the neural network model. The multi-client QoE fairness problem considers both the improvement of the QoE of clients and the achievement of the fair QoE.
- We trained the neural network model with a state-of-the-art reinforcement learning algorithm to generate the optimal policy for multi-client adaptive streaming. In training, network datasets with various conditions were used for generalizing the neural network model.
- We compared the proposed scheme with the existing schemes through simulation-based experiments. The experimental results show that the proposed scheme achieves better performance than the existing schemes in terms of the overall QoE of clients and the QoE fairness among clients.

The remainder of this paper is organized as follows. Section 2 presents related work on the QoE optimization of multi-client adaptive streaming and the reinforcement learning-based bitrate adaptation. In Section 3, we describe the design of the framework of the proposed scheme. Simulation-based experiments used to evaluate the proposed scheme are presented in Section 4, and we conclude the paper in Section 5.

## 2. Related Work

In this section, first we illustrate the background of DASH-based bitrate adaptation. Thereafter, we present several challenges of bitrate adaptation for multi-client adaptive streaming. Finally, we illustrate the application of reinforcement learning to adaptive streaming. Existing schemes utilizing reinforcement learning are also illustrated with respect to this.

### 2.1. DASH-Based Bitrate Adaptation

HTTP adaptive streaming standardized as DASH is the main scheme for video delivery over the Internet today. Through video transmissions based on HTTP, content providers can utilize the existing Content Delivery Networks (CDNs) at a low cost.

**Figure 1:**
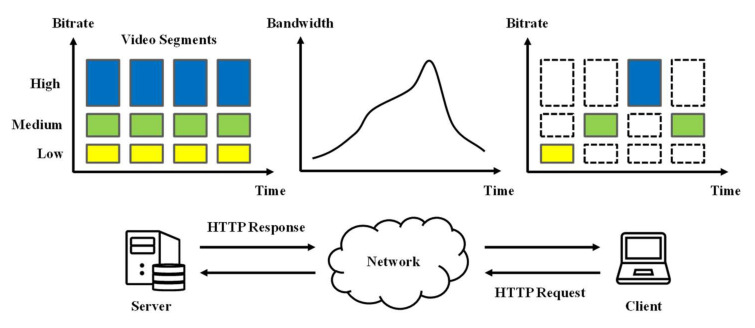
Concept of DASH-based bitrate adaptation.

In DASH, the videos are stored on the server as multiple segments in advance, each of which includes a part of video playback. The video segments are encoded at multiple discrete bitrates to support bitrate adaptation according to the time-varying network conditions. A higher bitrate indicates a higher quality and leads to a larger segment size. Figure 1 shows the concept of DASH-based bitrate adaptation. Once the video streaming begins, the client first demands a Media Presentation Description (MPD) file from the server. The MPD file includes segment information, such as bitrate, size, playback length, and address to request. Then, the client requests the video segments through its adaptation algorithm. This algorithm utilizes a variety of contexts, such as the measured available bandwidth and the current buffer level, to determine the bitrate of future segments. DASH-based bitrate adaptation improves the QoE of clients by matching the video bitrate with the conditions of clients [16]. However, the client-driven scheme of DASH does not consider the status of the other clients and tries to occupy the available bandwidth of the network as much as possible. This greedy characteristic causes bandwidth starvation of the other clients, leading to QoE degradation and unfair QoE. In this regard, it is challenging to achieve high overall QoE and high QoE fairness without considering multi-client competition.

### 2.2. Challenges for Bitrate Adaptation in Multi-Client Adaptive Streaming

The fundamental reason for performance degradation in multi-client adaptive streaming is the lack of coordination among clients. Each client recognizes the network conditions based on bandwidth measurements according to the segment throughput. The throughput-based measurement performs well when the number of clients is small, and the network conditions are stable. If there are many clients in the same network, leading to the worsening of multi-client competition, the segment request patterns of clients make accurate bandwidth measurement difficult [17]. Moreover, the dynamic network conditions burden the clients in determining the next bitrate, implying that the available bandwidth may change for various reasons in addition to the occupation of bandwidth by the other clients.

To solve this problem, a bitrate adaptation scheme considering the trade-offs be-tween reducing traffic volume and maintaining the QoE has been proposed [18]. Through a heuristic-based algorithm, this scheme determines the video bitrates of multiple clients that minimize unnecessary traffic and QoE degradation. The other approaches to improve the performance of multi-client adaptive streaming have focused on moving adaptation intelligence to servers or in-network elements [19,20,21,22]. The key idea of these approaches is to supervise clients in a centralized manner by aggregating the information of clients. This improves fairness as well as overall QoE. However, additional modifications are required for the server, the in-network elements, and the clients. This causes scalability problems in the adaptive streaming that handles multiple clients simultaneously. Thus, schemes moving adaptation intelligence to the mobile edge have emerged for efficient multi-client adaptive streaming [23,24,25,26]. In the middle of the server and the clients, the mobile edge collects the information of clients and the specific network condition, such as the channel status. The mobile edge determines the bitrate of video segments to be requested by jointly considering the QoE of clients, the resource utilization, and the fairness among clients. Compared with the assistance of the server or the in-network elements, the adaptive streaming schemes based on edge computing assistance can easily access the information of clients and channels. With minor modifications to the clients, these schemes support bitrate adaptation that does not violate the existing DASH standard. The proposed scheme utilizes edge computing to obtain this advantage and to manage multiple clients efficiently.

The existing solutions for multi-client adaptive streaming aim to achieve high levels of efficiency, stability, and fairness [27]. Improving the efficiency and the stability implies that the clients achieve high average quality, few quality variations, and a low re-buffering ratio. Moreover, improving the fairness indicates that all the clients request the same bitrate as far as possible (i.e., bitrate fairness). Bitrate fairness depends on the TCP mechanism of equally achieving the bandwidth among multiple users through congestion control. The clients may experience different QoE even if the same bitrate is allocated because the TCP mechanism is blind to the quality of user experiences. The QoE of clients is determined by the changes in requested bitrate and the degree of maintenance of seamless video playback by the clients. Therefore, allocating the same bitrate to the clients cannot satisfy the fair quality of user experiences. It is important to allocate the as much bandwidth as the client needs to improve the QoE of clients while achieving a fair QoE [28]. The adaptive streaming schemes considering QoE fairness have been researched in recent years [29,30]. These schemes formulate the joint optimization problem for overall QoE and QoE fairness. We model the optimization target of the proposed scheme in a similar way to this method. Moreover, we utilize reinforcement learning to adapt video bitrate delicately at the mobile edge, even in the case of high multi-client competition and high variance of the network conditions. In the next subsection, we discuss why applying reinforcement learning to adaptive streaming is effective and how this approach improves the performance compared with the existing heuristic-based schemes.

Among the existing schemes for multi-client adaptive streaming, we used ECAA as the comparison scheme to evaluate the performance [24]. ECAA adopts edge computing assistance to determine the video bitrates of multiple clients. However, ECAA considers bitrate fairness rather than QoE fairness. To allocate the same bitrates for all the clients, the bitrate adaptation is designed to represent the greedy-based algorithm. This heuristic algorithm achieves suboptimal performance in the network conditions with high variability. In addition to using the concept of edge computing assistance, the proposed scheme learns the policy for multi-client adaptive streaming through reinforcement learning. The policy generated by the neural network model improves the performance even in the network conditions with high variability. On the basis of the fair QoE approach, the proposed scheme makes the multiple clients in the same network utilize the bandwidth as much they need. This strategy leads to a fair and maximized QoE in multi-client environments with dynamic network conditions.

### 2.3. Reinforcement Learning-Based Adaptive Streaming

The adaptive streaming clients recognize the current state after the video segments are completely received. The bitrate of the next video segment is determined according to the states derived from the interactions between the server and the clients. In other words, the QoE optimization in adaptive streaming means finding the optimal bitrate selection policy in the current state. Reinforcement learning has the objective of maximizing the future cumulative rewards for a given environment. The agent learns a sequential policy to satisfy this goal. Therefore, the QoE optimization of adaptive streaming is appropriately modeled through reinforcement learning.

The existing adaptive streaming schemes based on reinforcement learning consider the following challenges. First, the network conditions fluctuate over time and vary across the environments of clients. This complicates bitrate adaptation, as the input contexts have different importance when adapting the video bitrate. In the time-varying links with high and stable average bandwidth, the best choice for QoE optimization is to maintain a high bitrate even though the buffer level becomes low. In the opposite case, the clients need to consider the buffer level first when determining the bitrate of video segments to reduce the re-buffering ratio. Second, the bitrate adaptation should strike a balance among the various objectives, such as maximizing the average quality, minimizing the quality variations, and reducing the re-buffering events. These objectives are in conflict with each other. For example, requesting video segments with high bitrate maximizes the average quality, but the re-buffering ratio increases. Changing the bitrate at the right time improves the fairness, but the quality variations increase, and the buffer level of clients becomes unstable. Moreover, the preferences for the factors that influence QoE may vary significantly across users [31,32]. The next challenge is the cascading effect of bitrate selection. For example, selecting a high bitrate may force clients to request the subsequent video segments with low bitrates to avoid re-buffering events. Finally, the bitrate decision is not fine-grained, due to the limitation of the available video bitrates. In many cases, the available bandwidth does not fit the video bitrates. The bitrate adaptation algorithm in these cases should decide the QoE influencing factor that must be considered first; thus, the QoE optimization for various network conditions becomes difficult.

The reinforcement learning-based adaptive streaming schemes enable the agents to experience various network conditions through learning [33,34,35,36,37]. The agents generate and improve the policy in the directions that lead to better results. Unnecessary parameter tuning is not required thanks to this trial-and-error approach. Therefore, the bitrate adaptation based on reinforcement learning has better performance than the heuristic-based schemes in various network conditions. The proposed scheme utilizes these features of reinforcement learning, but the goal of learning is to optimize both the overall QoE and QoE fairness. In contrast to the existing schemes that focus on a single client, we designed the framework of the proposed scheme in terms of multi-client adaptive streaming.

We used Pensieve as the comparison scheme to evaluate the performance, choosing it from among the existing schemes that are based on reinforcement learning [33]. Pensieve adopts reinforcement learning to generate the policy, achieving high QoE performance even in dynamic network conditions. However, Pensieve focuses on bitrate adaptation for a single client. There is a lack of consideration for the multi-client competition and QoE fairness among clients. Pensieve aims to learn the policy maximizing the individual QoE. The trained model for Pensieve is deployed to the server. This imposes additional modifications to the server and the clients to support the centralized bitrate adaptation by the neural network model. Unlike Pensieve, the proposed scheme jointly considers QoE fairness among clients and the individual QoE when determining the bitrates of the multiple clients. Moreover, the concept of edge computing assistance is adopted to manage the multiple clients and support DASH-based bitrate adaptation in the middle of the server and the clients. In the proposed scheme, the states, the reward, and the training method used to learn the policy for achieving the optimal multi-client QoE fairness are designed differently compared with those of Pensieve. On the basis of these differences, the proposed scheme improves both the QoE fairness among clients and the individual QoE in multi-client environments with dynamic network conditions.

## 3. Framework Design

This section describes the basic assumptions and framework of the proposed scheme. We formulate the multi-client QoE fairness problem by using the concept of MDP and define various parameters and approaches required to train the neural network model.

**Figure 2:**
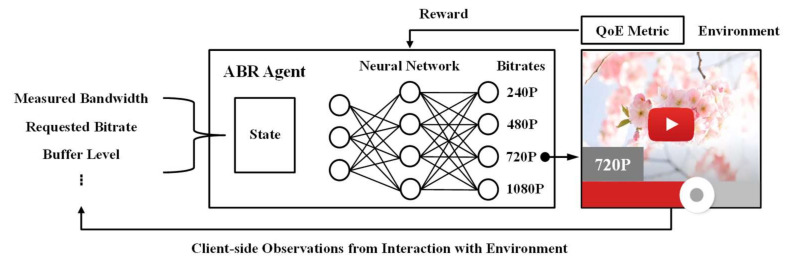
Concept of applying reinforcement learning to bitrate adaptation.

Figure 2 shows how reinforcement learning is applied to bitrate adaptation. After the states are passed to the Adaptive Bit Rate (ABR) agent, the agent performs an action to select the bitrate. The agent receives the resulting rewards from the environment. The reward is determined as the instant QoE for the video segments received by the clients. To design the framework of the proposed scheme, we need to concretize the states, action, reward, neural network model, and training methodology.

Before presenting the details, we illustrate the basic assumptions of the proposed scheme. All clients request the same video from the server. The network conditions perceived by the clients are derived from multi-client competition and channel variability. To this end, we ignore the effect of the slow start of TCP in simulations for training and testing the neural network model. The slow start makes gathering accurate simulation difficult because it associates network throughput with the adaptation algorithm being used. For example, the algorithm filling the buffer frequently and quickly causes the slow start phases; thus, the bandwidth utilization of clients is degraded. Next, we assume that device heterogeneity among clients does not exist; hence, each client expects the same video quality if the network conditions are similar. Finally, the clients periodically report their observations to the mobile edge. One solution for the reporting process is to add the QoE-related information to the HTTP header of the segment requests. In the proposed scheme, the clients use this method to communicate with the mobile edge. The details of capturing the segment requests at the mobile edge and modifying the request information are beyond the scope of this study. The proposed scheme ignores the computation delay from capturing and modifying the segment requests of clients at the mobile edge. In the simulations, the packet loss of links is not considered, and the delay of communication links is fixed.

### 3.1. State

The agent generates and improves the policy by considering the input states. Therefore, we should carefully determine the observations to be used as the states. If the state space is extremely small, the agent experiences a loss of information in learning, leading to limitations in policy improvement. It is difficult to train the agent to generate the optimal policy for the given environments when the state space is extremely large. For the policy improvement to move in the correct direction, the states should be matched well to the optimization goal. The goal of the proposed scheme is to generate the policy that improves both QoE fairness among clients and the individual QoE. To this end, the states should be related to the factors influencing QoE in bitrate adaptation. We define the state space at time step t as $S_{t}=\{{\vec{x}}_{t},\;{\vec{d}}_{t},\;{\vec{z}}_{t},\;o_{t},\;v_{t}\}$, where ${\vec{x}}_{t}$ is the vector of past segment throughput and ${\vec{d}}_{t}$ is the vector of past segment download time. ${\vec{z}}_{t}$ means the next segment size for video bitrate levels. $o_{t}$ is the current buffer level of clients and $v_{t}$ is the last requested bitrate. The segment throughput and the segment download time indicate the status of the network where the clients stream videos. The segment sizes for different bitrates vary even in the same video according to the complexity of the video frames. By considering this, the agent predicts how much bandwidth is required to receive the video segments. The agent recognizes the risk of video re-buffering by considering the current buffer level. Moreover, the agent needs to know the current bitrate to control the smoothness of video streaming and determine the maximum bitrate, satisfying the available bandwidth.

Unlike Pensieve [33], we do not use the number of segments left as the states. This is because the optimization goal of the proposed scheme is to improve both the overall QoE and QoE fairness. In fact, the number of segments left helps the agent to maximize the individual QoE of clients, but this behavior hinders achieving a fair QoE. The agent in Pensieve tends to determine the high bitrate at the end point of the video session. Therefore, the other clients sacrifice their bandwidth and experience severe network congestion. The network congestion degrades the QoE of clients and the bandwidth utilization. The proposed scheme does not consider the number of segments left, but the agent learns the optimal policy by using the rewards based on multi-client QoE fairness.

### 3.2. Action

The policy is determined by the states and the corresponding actions. The action taken by the agent affects the next states and the resulting rewards. The proposed scheme defines the action space at time step t as $A_{t}=\left\{w_{1},\;w_{2},\;\ldots ,\;w_{L}\right\}$. L is the number of video bitrate levels, and $w_{l}$ is the video bitrate with a level l. Each action refers to the bitrate selection for the upcoming segments. The policy generated by the neural network model plays a role in determining the bitrates for multiple clients. For this reason, the bitrate selection for the upcoming segments is defined as the action. To simplify the problem space of the proposed scheme, we assume that the number of video bitrate levels is fixed. Moreover, we set the fixed values for the playback length of video segments and the number of video segments in training and testing the neural network model. The output layer of the model has multiple neurons, each of which indicates the selection probability of video bitrates. According to the determined action, the agent at the mobile edge adjusts the bitrate of video segments in real time. The agent determines the next action randomly when training the neural network model. In testing, the agent determines the action with the highest selection probability as the next action.

### 3.3. Reward

Setting the rewards is a critical challenge when generating an effective policy in reinforcement learning. First, we determine the basic form for the QoE of clients. Many factors influence the QoE of clients, such as the video quality, the quality variations, and the video re-buffering. The existing schemes to optimize the QoE of clients generally quantify the QoE value as a linear combination of these influencing factors.

(1) $$QoE_{i}=q\left(b_{i}\right)-\delta \left|q\left(b_{i}\right)-q\left(b_{i-1}\right)\right|-\rho T\left(b_{i}\right)$$

where $QoE_{i}$ is the QoE value for the ith video segment, $q\left(b_{i}\right)$ is the quality of the ith video segment with bitrate $b_{i}$, and $T\left(b_{i}\right)$ is the video re-buffering time after receiving the ith video segment with bitrate $b_{i}$. δ and ρ are the weight parameters used to control the impact of quality variations and video re-buffering, respectively. There are many methods for determining the relationship between video quality and video bitrate. We adopt the simplest method that regards video bitrate itself as video quality.

We extend the QoE value by considering the multi-client QoE fairness to concretize the rewards. If the QoE value is low, then the client needs to receive more bandwidth to improve their QoE. Contrary to this point, if the QoE value is high, then the client needs to allow the other clients to improve their QoE by sacrificing some bandwidth. The clients do not have the same QoE, even if they use the same bandwidth. Achieving a fair QoE means that all the clients use the bandwidth as much they need to improve or maintain their QoE. We formulate the utility value as a combination of individual QoE and QoE deviations among clients. The value of the multi-client QoE fairness is calculated as below.

(2) $$r_{t}=QoE_{i}-\left|\varepsilon \right|\frac{\left|QoE_{i}-QoE_{avg}^{k}\right|}{QoE_{max}-QoE_{min}},\;\;\forall \;k=1,\;2,\;\ldots ,\;N$$

where $r_{t}$ is the value of the rewards at time step t and ε is the weight parameter that combines the individual QoE with the QoE deviations. $QoE_{avg}^{k}$ is the average QoE of all the clients, except for client k. N is the total number of clients. $QoE_{max}$ and $QoE_{min}$ are the maximum and minimum values in the QoE of clients, respectively. These values are updated whenever the clients receive new video segments. If the current QoE deviations are high, the agent needs to improve QoE fairness. The agent needs to generate the policy that leads to the bitrate achieving high levels of quality, smoothness, and stability as much as possible for each client when the current QoE deviations are low. The weight parameter controls the impact of individual QoE and QoE deviations in the rewards. Therefore, it is important to set the value of this parameter appropriately. The range of the weight parameter should be between $QoE_{min}$ and $QoE_{max}$ to match the dimensions with the QoE value. We set the value of the weight parameter as the minimum value in the QoE of clients.

Let us explain an example to understand how the impact of individual QoE and QoE deviations varies according to the value of the weight parameter. Immediately after the video streaming starts, the buffer level is low and there is insufficient information on how to adapt the bitrate of future video segments. The risk of video re-buffering and the quality variations increase in this case; hence, the QoE of clients becomes low. By considering this situation, the agent tries to learn the policy that reduces the QoE deviations in the initial phases of video streaming. This policy means that the clients select the low bitrate at the initial phases of video streaming and increase the bitrate gradually. While the video streaming continues, the QoE of clients becomes high if the agent has learned the policy with the strategy that we mentioned before. In this case, the agent tries to learn the policy that maintains the high individual QoE and changes the bitrate at the right time. Thanks to this strategy, the QoE of other clients does not suffer from abrupt changes in the individual QoE. Furthermore, the proposed scheme does not change the bitrate unnecessarily if a sufficient buffer exists, reducing the instability in the buffer and the bitrate variations.

To formulate the multi-client QoE fairness problem by using the MDP, we have illustrated the basic elements, such as the states, the action, and the rewards. In the following subsections, we present the architecture of the neural network model, the training algorithm used to generate the policy, and the multi-agent approach for considering how multiple clients compete for limited bandwidth.

### 3.4. Neural Network Model

To learn the optimal policy, it is important to construct the architecture of the neural network model appropriately. This means that the neural network model should be sufficiently large to generate an elaborate policy, and the complexity of the architecture should be low to avoid the burden of training. We adopt a simple architecture using convolution layers and fully connected layers. The proposed scheme uses a training algorithm based on the actor–critic approach. This approach trains an actor network and critic network simultaneously [38]. The actor network plays a role in determining the action when the states are given. The critic network evaluates the current value of the states and passes this information on to the actor network to assist its parameter updates. The existing training algorithms have the problem that the learning process is time-consuming and learning results are not converged. This is due to the high variance in learning when the training episode is long, or the optimization target is complex. The actor–critic algorithm reduces the variance in learning by utilizing the complementary training process.

**Figure 3:**
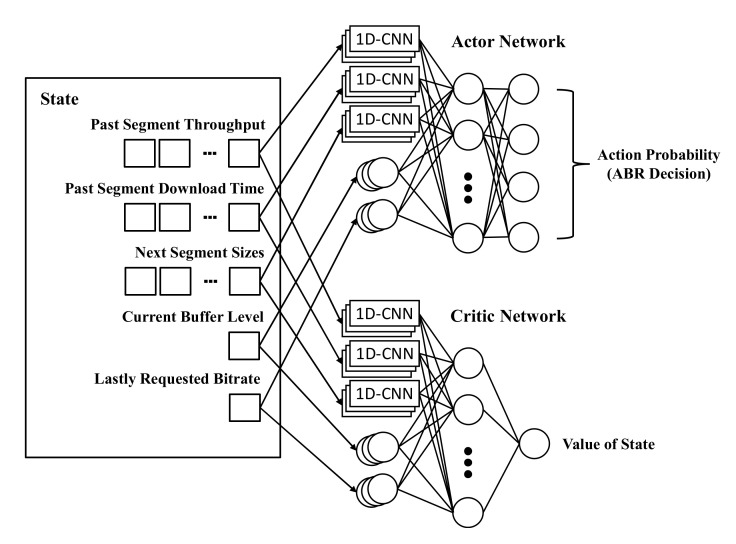
Architecture of actor network and critic network for generation of the policy.

Whenever downloading each video segment, the agent passes the states into the neural network model. Figure 3 shows how the input states are passed to the neural network model, and how the outputs of the actor network and critic network are determined. Single inputs, such as the current buffer level and the lastly requested bitrate, are passed to the normal neurons. Multiple inputs, such as the past segment throughput, the past segment download time, and the next segment sizes, are passed to the 1D Convolutional Neural Network (1D-CNN) neurons. The 1D-CNN layer is commonly utilized to extract the features for multiple inputs. The extracted features are transferred to the fully connected layers, used as the hidden layers of the neural network model. According to the parameters of the hidden layers, the output layer of the actor network calculates the selection probability for the video bitrates. The high selection probability means that the agent improves the rewards when determining the corresponding bitrate. The output layer of the critic network calculates the current value of the states. This value is represented as an integer and is utilized by the actor network to update its parameters.

The agent selects the next action based on the policy as according to the probability distribution over the actions. After the action is performed, the simulated environment of the proposed scheme provides rewards for the received video segments to the agent. The proposed scheme maximizes the expected cumulative rewards by using a policy gradient method [39]. The basic idea of the policy gradient method is to estimate the gradient of the expected total rewards by observing the trajectories of executions from the policy. The agent updates the parameters of the neural network model, making the neural network model select the action with high rewards frequently. The gradient of the expected cumulative rewards is calculated as follows, by considering the policy parameters.

(3) $$\nabla_{\theta }E_{\pi_{\theta }}\left[\sum_{t=0}^{\infty }\gamma^{t}r_{t}\right]=E_{\pi_{\theta }}\left[\nabla_{\theta }\;log\;\pi_{\theta }\left(s,\;a\right)A^{\pi_{\theta }}\left(s,\;a\right)\right]$$

where θ represents the policy parameters, γ is the discount factor controlling the impact of future rewards, and $r_{t}$ is the value of the rewards at time step t. $\pi_{\theta }\left(s,\;a\right)$ is the policy at state s with action a, and this value is represented as the probability with a range from 0 to 1. $A^{\pi_{\theta }}\left(s,\;a\right)$ is the advantage function that implies the difference in the expected rewards when we select the action a at the state s deterministically, compared with the expected rewards from policy $\pi_{\theta }$. In other words, the value of the advantage function indicates how much better the specific action is than the average action taken by the policy.

The neural network model is used to represent the policy with a manageable number of adjustable parameters. Therefore, updating the parameters for the actor network and critic network is important when generating the policy that can maximize the future rewards of the agent. First, each update of the parameters of the actor network is represented as follows, according to the policy gradient method.

(4) $$\theta \leftarrow \theta +\alpha \sum_{t}\nabla_{\theta }\;log\;\pi_{\theta }\left(s_{t},\;a_{t}\right)A\left(s_{t},\;a_{t}\right)$$

where θ represents the parameters of the actor network that are equal to the policy parameters we mentioned before, which are used to formulate the gradient of the expected cumulative rewards. α is the learning rate used to update the parameters of the actor network. $s_{t}$ and $a_{t}$ are the states and the action at time step t, respectively. The intent of this update rule is as follows. $\nabla_{\theta }\;log\;\pi_{\theta }\left(s_{t},\;a_{t}\right)$ specifies how to change the parameters of the actor network to increase the probability of selecting action $a_{t}$ at states $s_{t}$. $A\left(s_{t},\;a_{t}\right)$ plays a role in accelerating the parameter update to obtain the empirically better returns. The size of the update step depends on the value of the advantage function. As an unbiased estimate of $A^{\pi_{\theta }}\left(s,\;a\right)$, the value of the advantage function is computed over the episodes the agent experiences.

We need to estimate $v^{\pi_{\theta }}\left(s\right)$ first to utilize the value of the advantage function in updating the parameters of the actor network. The value function refers to the expected total rewards following policy $\pi_{\theta }$ when starting from states s. The key function of the critic network is to learn how to predict the value function from the observed rewards. We adopt a standard Temporal Difference (TD) method to update the parameters of the critic network [40].

(5) $$\theta_{v}\leftarrow \theta_{v}-\alpha^{\prime }\sum_{t}\nabla_{\theta_{v}}{\left(r_{t}+\gamma V^{\pi_{\theta }}\left(s_{t+1};\;\theta_{v}\right)-V^{\pi_{\theta }}\left(s_{t};\;\theta_{v}\right)\right)}^{2}$$

where $\theta_{v}$ is the parameters of the critic network and $\alpha^{\prime }$ is the learning rate used to update the parameters of the critic network. $V^{\pi_{\theta }}\left(s_{t};\;\theta_{v}\right)$ is the output of the critic network, and this value is utilized as the estimate for $v^{\pi_{\theta }}\left(s\right)$ at time step t. It is well known that the difference in the value functions calculated using the TD method can be used as the advantage function [41]. Note that the critic network merely participates in updating the parameters of the actor network. After the training process for the actor network and the critic network ends, the agent uses only the actor network to perform bitrate adaptation.

For discovering a good policy, we should ensure that the agent explores the action space sufficiently during training. To this end, adding an entropy regularization term to the parameter update rule of the actor network is a practical solution. We modify Equation (4) by considering this scheme as follows.

(6) $$\theta \leftarrow \theta +\alpha \sum_{t}\nabla_{\theta }\;log\;\pi_{\theta }\left(s_{t},\;a_{t}\right)A\left(s_{t},\;a_{t}\right)+\beta \nabla_{\theta }H\left(\pi_{\theta }\left(\cdot |s_{t}\right)\right)$$

where $H\left(\pi_{\theta }\left(\cdot |s_{t}\right)\right)$ is the entropy for policy $\pi_{\theta }$ in states $s_{t}$, encouraging the explorations by updating θ in the direction with higher entropy. β is the entropy weight that controls the degree of explorations. The entropy weight is set to a large value at the beginning of training and decreases gradually over time. The agent learns a policy with low variance by controlling the entropy weight.

### 3.5. Multi-Agent Training Methodology

The proposed scheme aims to optimize the performance of multi-client adaptive streaming. Therefore, we need to determine a training methodology that considers multi-client competition. From this perspective, the multi-agent training approach can be used, which is effective in the case where multiple agents should share their situations with each other and there exists heterogeneity among the agents [42,43]. Moreover, this approach helps to speed up the training process by distributing the aggregated information for the parameter updates to all the agents.

**Figure 4:**
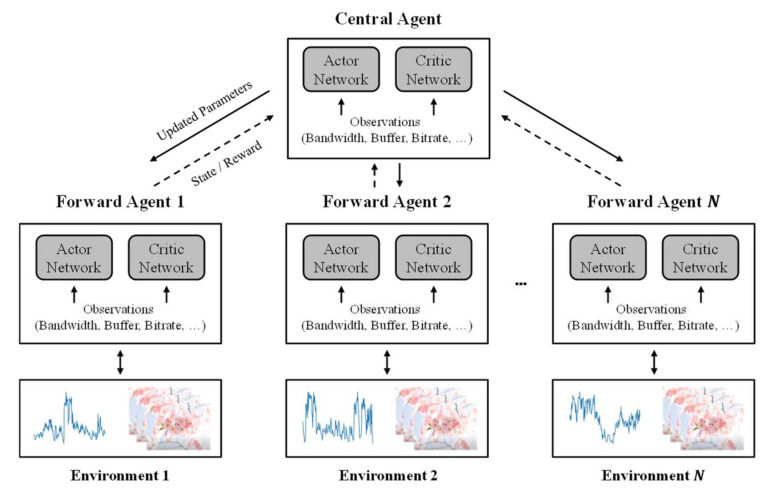
Multi-agent training process used to train the neural network model.

Figure 4 shows the multi-agent training process used to train the neural network model. This process runs multiple agents simultaneously. The forward agents are configured to experience a different set of input conditions, such as the changes in the available bandwidth. Herein, we assume that the input network traces (i.e., bandwidth logs) determine the changes in the available bandwidth for each agent due to the multi-client competition and the channel variability. The forward agents collect their states, actions, and rewards during each episode, represented by one bandwidth log. The states are derived from the segment throughput, the requested bitrate, and the buffer level, which are experienced by the forward agents for the given available bandwidth. The action and the rewards are derived from the bitrate adaptation by the forward agents. All the forward agents have their own actor network and critic network, and the collected information is transferred to the central agent that plays a key role in updating the parameters of the neural network model. The central agent also has its own actor network and critic network and updates the parameters of the neural network model by using the policy gradient-based rules. The learning objective of the proposed scheme is to generate the policy that achieves the fair and maximized QoE. To this end, the reward based on multi-client QoE fairness is calculated. The central agent calculates the reward by using the aggregated information of the forward agents. In other words, the forward agents share their information about multi-client adaptive streaming through cooperation with the central agent. The parameter updates of the neural network model are completed by the central agent. The updated parameters are then copied to all the forward agents. Different from the single agent training, this approach, based on centralized training and decentralized execution, enables the neural network model to learn the policy by utilizing the knowledge of multi-client adaptive streaming. Such procedures are repeated until the training of the neural network model ends.

It is important to set the appropriate number of training agents for efficient multi-agent training. In general, the content server provides video segments to many clients. However, only a few dozen clients are handled momentarily by the content server. First, we set the maximum number of clients performing bitrate adaptations simultaneously. Thereafter, we determine the number of training agents to be the same as the maximum number of clients, such that the agents experience the most dynamic network conditions in the given environments. This value is also used in the experiments as the maximum number of clients to evaluate the performance of the proposed scheme by considering multi-client competition.

Each client cannot recognize the changes in network conditions due to the multi-client competition if there is no proxy or mobile edge. The agents share information about the individual QoE and the current QoE deviations. The central agent in multi-agent training plays a role in managing multiple agents by using the global knowledge for bitrate adaptation, similar to the mobile edge for adaptive streaming. The multi-agent training is conducted offline based on simulations, and the neural network model is trained during a fixed number of epochs. We deploy the trained model to the mobile edge to perform multi-client bitrate adaptation with edge computing assistance. Once the video streaming starts, the mobile edge generates multiple instances of the trained model according to the number of clients. The outputs of the instances denote the video bitrate that improves the rewards in the given states. The mobile edge adjusts the video bitrate of the clients to the outputs of the instances.

## 4. Performance Evaluation

In this section, we first illustrate the details of implementing the neural network model. Then, we present the network datasets utilized in training and testing the neural network model, the existing schemes for performance comparisons, and the evaluation metrics. The evaluation results are presented to compare the overall QoE and the QoE fairness of the proposed scheme and the existing schemes. Moreover, we analyzed the bitrate changes in each scheme during video streaming.

### 4.1. Implementation

TensorFlow was used to implement the neural network model [44]. To utilize the implemented model in training and testing, we leveraged the API of TFLearn deep learning library [45]. The 1D-CNN layers of the actor network have 128 filters. The size of each filter is four and the stride is one. The agent passes the past segment throughput, the past segment download time, and the next segment sizes to the 1D-CNN layers and subsequently extracts the features of the inputs. The extracted features are aggregated with other inputs in the hidden layer that uses 128 neurons. In the output layer, the number of neurons is equal to the number of available video bitrates. Each neuron uses a softmax function for the output. The architecture of the critic network is the same as that of the actor network, but the output is generated by a linear neuron with no activation function. We summarize the parameter description for the multi-agent training of the proposed scheme, as shown in Table 1.

We set the number of multiple inputs passed to the 1D-CNN layers to eight and the discount factor to 0.99 while training the neural network model. The value of the discount factor determines the impact of the future steps on the current action. Setting the value of the discount factor to 0.99, the agent selects the current action by considering 100 future steps. The learning rates for the actor network and the critic network are set to 0.0001 and 0.001, respectively. The learning rate of the actor network is determined to be lower than that of the critic network to avoid unstable behaviors during training. Moreover, we control the value of the entropy weight, making it decay from 5.0 to 0.1 over a total of 100,000 training epochs, to derive the parameter update of the neural network model in the correct direction. We need to set the values of the weight parameters used in the QoE of clients to measure the multi-client QoE fairness. We calculate the rewards by adopting the values of the weight parameters used in reference [10]. The maximum number of clients is set to 20, such that the training agents experience the sufficiently dynamic network conditions in the given environments.

Ideally, the neural network model should be trained by emulating the actual environments of adaptive streaming. However, this method is slow because the agent must wait until all the video segments are downloaded and to explore the current environment before updating the parameters of the neural network model. The proposed scheme uses a simple simulator to model the bitrate dynamics of adaptive streaming. The simulator measures the segment download time based on the video bitrate of the segments and the available bandwidth of the network traces. The simulator then drains the buffer as much as the current download time allows and adds the playback length of the segments to the buffer. While simulating the adaptive streaming for the given network traces, the simulator keeps track of the video re-buffering events. When the buffer cannot accommodate video data, the simulator pauses the request process for 500 ms before retrying the segment requests. After downloading the video segments, the simulator passes the current states to the agent, and the agent determines the next action. The neural network model experiences hundreds of hours of adaptive streaming in tens of minutes by using this simulator.

### 4.2. Experimental Setup

**Figure 5:**
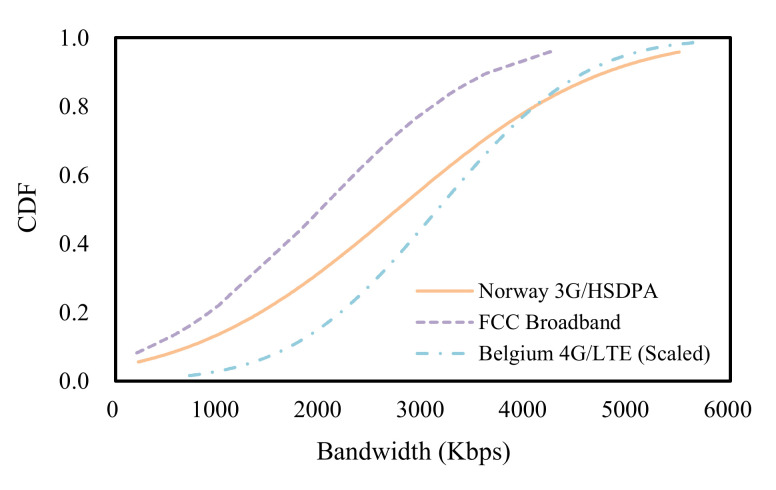
Bandwidth of network datasets.

We use several network datasets to evaluate the performance of the proposed scheme and the existing schemes. These datasets include the broadband dataset provided by FCC, the 3G/HSDPA mobile dataset collected in Norway, and the 4G/LTE mobile dataset collected in Belgium [46,47,48]. The FCC dataset has over 1 million bandwidth logs, each of which records the average bandwidth over 2100 s, at 5 s granularity. The 3G/HSDPA dataset comprises 30 min of bandwidth measurements of mobile devices streaming videos while in transit (e.g., on buses, trains, etc.). The 4G/LTE dataset consists of bandwidth measurements of mobile devices receiving files in the LTE network. We generated a total of 3000 bandwidth logs by combining these datasets, and each log has a playback duration of 320 s. Especially for the 4G/LTE dataset, we scaled the value of the bandwidth in the logs to ensure compatibility with the FCC dataset and the 3G/HSDPA dataset. Figure 5 shows the bandwidth of the network datasets used in training and testing the neural network model. The network datasets have different average bandwidths and different bandwidth variations. To avoid the trivial bitrate selection of the agents during training and testing the neural network model, we only used the bandwidth logs where the minimum bandwidth is above 0.2 Mbps and the maximum bandwidth is less than 6 Mbps. Unless otherwise noted in the experiments, a random sample of 80% of the bandwidth logs was used as the training set and the remaining 20% was used as the testing set.

**Figure 6:**
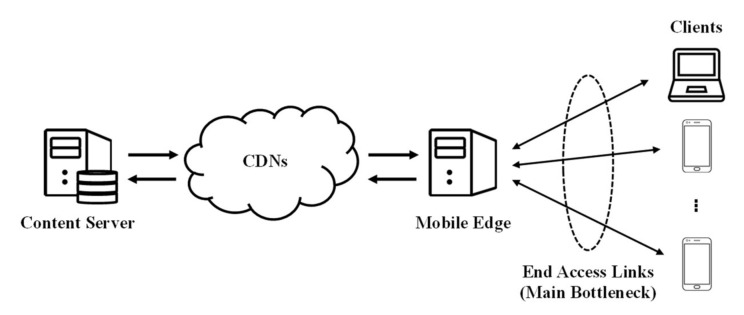
Networking model for multi-client adaptive streaming in edge computing environments.

For the experiments, we set up the networking model for multi-client adaptive streaming with edge computing environments, as shown in Figure 6. We did not implement an explicit communication process between the content server and the mobile edge to simplify the simulations. The mobile edge obtains the video segments and the MPD file from the content server after the video streaming starts, playing a role as the content server in the end access links (i.e., the main bottleneck links in the multi-client competition). Therefore, we implemented only the parts of the mobile edge and the clients on the basis of the simulation environments.

We used the existing schemes that utilize edge computing assistance or reinforcement learning in the experiments. First, the scheme, named ECAA, adopts edge computing assistance for efficient multi-client bitrate adaptation [24]. ECAA suggests not only greedy-based bitrate selection, but also edge server allocation. In the experiments, we do not consider edge server allocation when evaluating the performance in terms of multi-client adaptive streaming. We set the various parameters of ECAA equal to those in reference [24]. Second, Pensieve applies reinforcement learning to adaptive streaming for QoE optimization [33]. This scheme uses multi-agent training, but both the rewards the agent aims to optimize and the number of training agents are different from those in the proposed scheme. For fair comparison, the same bandwidth logs are used in training and testing the neural network models for Pensieve and the proposed scheme. Moreover, we use the same weight parameters to model the QoE of clients.

Reasonable metrics should be defined to evaluate how much the QoE of clients is improved and how fair the QoE distribution among clients is. To this end, we measure the overall QoE and QoE fairness. The overall QoE is the average value of all the clients’ QoE for the streaming sessions. We first calculate the average QoE of clients for each streaming session as follows.

(7) $$QoE=\frac{1}{M}\left(\underset{\begin{array}{c} Bitrate\;\\ Utility \end{array}}{\underbrace{\sum_{i=1}^{M}q\left(b_{i}\right)}}-\underset{\begin{array}{c} Smoothness\;\\ Penalty \end{array}}{\underbrace{\delta \sum_{i=1}^{M}\left|q\left(b_{i+1}\right)-q\left(b_{i}\right)\right|}}-\underset{\begin{array}{c} Rebuffering\;\\ Penalty \end{array}}{\underbrace{\rho \sum_{i=1}^{M}T\left(b_{i}\right)}}\right)$$

where QoE is the client’s QoE, averaged by the number of the requested video segments. M is the number of video segments requested during the streaming session and is determined to be a fixed value in training and testing the neural network model. The value calculated using the above equation is averaged again by the number of clients to measure the overall QoE. We also measure the individual QoE components to better understand the gains of the proposed scheme. These components are indicated as the bitrate utility, the smoothness penalty, and the re-buffering penalty.

The QoE fairness refers to how equally the QoE of clients is distributed. We adopt Jain’s fairness index to measure QoE fairness [49]. This index calculates the degree of deviations of the target value by considering the number of targets. In the worst case, the value of Jain’s fairness index is the ratio of 1 to the total number of targets, and 1 in the best case. The value of Jain’s fairness index is calculated for an arbitrary target as follows.

(8) $$J\left(x_{1},\;x_{2},\;\cdots ,\;x_{n}\right)=\frac{{\left(\sum_{j=1}^{n}x_{j}\right)}^{2}}{n\sum_{j=1}^{n}x_{j}^{2}}$$

where $J\left(x_{1},\;x_{2},\;\cdots ,\;x_{n}\right)$ is the value of Jain’s fairness index for the target value x, and n is the number of targets. We use the QoE of clients as the target value of Jain’s fairness index. The instant QoE for the video segments is calculated first, and then the QoE fairness is determined.

Before presenting the experimental results for the proposed scheme and the existing schemes, we illustrate the configuration of the video streamed to the clients, the evaluation scenarios, and the other factors. We use the Envivio-Dash3 video from the DASH-246 JavaScript reference player [50]. This video is encoded by the H.264/MPEG-4 codec at bitrates in {300, 750, 1200, 1850, 2850, 4300} kbps. Moreover, the Envivio-Dash3 video is divided into 48 segments, and the playback length of this video is 193 s. This means that each segment has approximately 4 s of video playback. We evaluate the performance of the proposed scheme and the existing schemes by changing the number of clients to 5, 10, 15, and 20. The clients experience different network conditions for the bandwidth logs randomly selected from the testing set. The maximum buffer level of clients is 60 s. Unless otherwise stated, all the experimental results are the average value for a total of 25 runs.

### 4.3. Results

**Figure 7:**
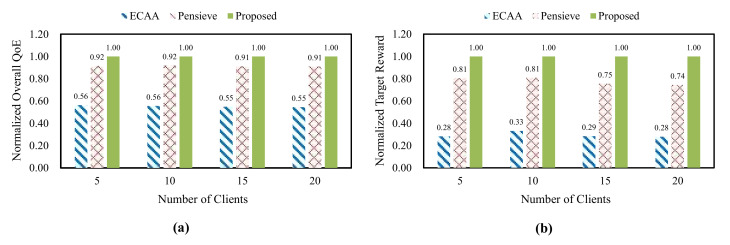
Overall QoE and target reward according to the number of clients, (**a**) normalized overall QoE, (**b**) normalized target reward.

According to the evaluation scenarios illustrated in the previous subsection, we first measured the overall QoE and the target reward for the proposed scheme and the comparison schemes. Figure 7 shows the results of the overall QoE and the target reward, which are measured by changing the number of clients. The target reward is the optimization target of the proposed scheme. By measuring the target reward for all the schemes, we compare how effectively each scheme balances between improving the individual QoE and reducing the QoE deviations. We normalized the values of the overall QoE and the target reward. The proposed scheme achieves the highest overall QoE compared with the existing schemes. This results from the optimization process that maximizes multi-client QoE fairness. Determining the adaptation strategy by considering the impact of QoE deviations on the individual QoE assists the clients in improving their QoE even with multi-client competition. As a result, the proposed scheme achieves the highest target reward.

**Figure 8:**
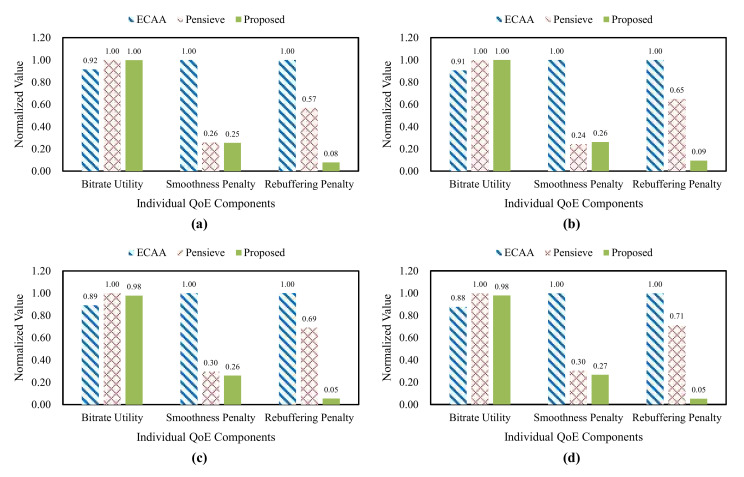
Comparisons of the individual QoE components according to the number of clients, (**a**) 5 clients, (**b**) 10 clients, (**c**) 15 clients, (**d**) 20 clients.

To analyze the reason behind the QoE improvements, we measured the individual QoE components for all the schemes according to the number of clients, as shown in Figure 8. We normalized the values of the individual QoE components. The proposed scheme achieves the lowest re-buffering penalty, regardless of the number of clients. Moreover, the proposed scheme achieves the highest value in the bitrate utility or the value like that of Pensieve. The proposed scheme achieves the lowest value in the smoothness penalty or the value like that of Pensieve. To improve QoE fairness, the optimization strategy of the proposed scheme sacrifices the average quality or the quality smoothness in some cases. The proposed scheme achieves the higher or similar QoE compared with Pensieve, although the optimization strategy has these characteristics. Especially in the re-buffering penalty, the proposed scheme outperforms the existing schemes for all the cases of changes in the number of clients. The proposed scheme maintains the client’s buffer stably, without abrupt bitrate changes, achieving a high bitrate utility and a low smoothness penalty.

Table 2 shows the quantified values of the overall QoE and the individual QoE components. These values are calculated by using the average for all the cases of changing the number of clients. The overall QoE of the proposed scheme is about 81% and 9% higher than that of ECAA and Pensieve. The bitrate utility of the proposed scheme is about 10% higher than that of ECAA and about 2% lower than that of Pensieve. Next, the smoothness penalty of the proposed scheme is about 74% lower than that of ECAA and about 7% lower than that of Pensieve. For the re-buffering penalty, which is significantly different from the existing schemes, the proposed scheme is about 93% lower than that of ECAA and about 89% lower than that of Pensieve. The proposed scheme improves the overall QoE, while minimizing the performance loss of the individual QoE components.

**Figure 9:**
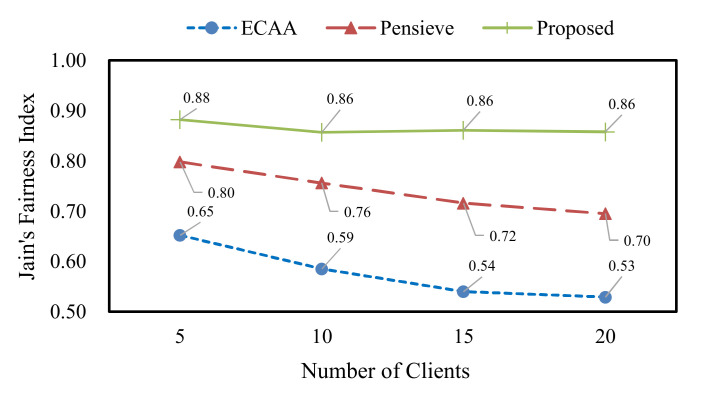
QoE fairness according to the number of clients.

Figure 9 shows the results of QoE fairness, which were measured by changing the number of clients. When the number of clients increases, the QoE fairness decreases due to the excessive multi-client competition. Nevertheless, compared with ECAA and Pensieve, the proposed scheme achieves the highest value in QoE fairness.

**Figure 10:**
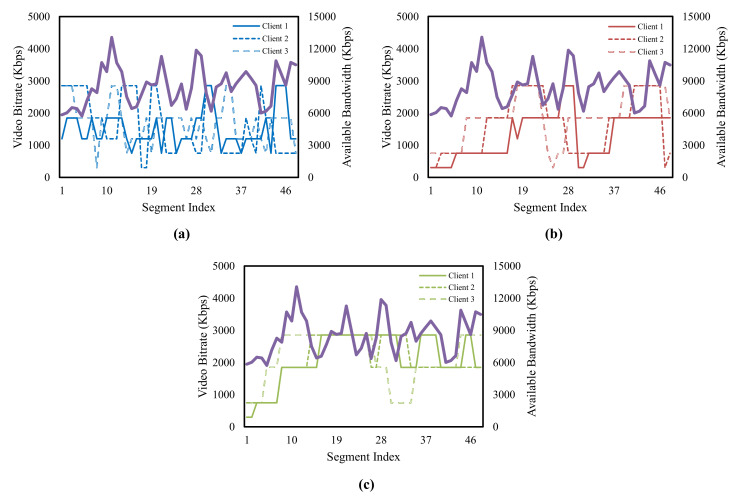
Comparisons of the bitrate changes of each scheme, (**a**) ECAA, (**b**) Pensieve, (**c**) Proposed.

As shown in Figure 10, we compared the bitrate changes for each scheme. For brevity, we present the results of the bitrate selection for the case where the number of clients is five and analyze the results of three clients that were randomly chosen. The available bandwidth in the figure means the total available bandwidth experienced by the three clients. The greedy-based bitrate selection algorithm of ECAA tends to determine the high video bitrate from the beginning of the streaming session. This causes instability in the buffer so that the clients change the video bitrate abruptly to avoid re-buffering. The abrupt changes in the video bitrate cause high QoE deviations and degrades the bandwidth utilization of the clients. The reinforcement learning-based bitrate selection of Pensieve stably controls the video bitrate that is requested compared with ECAA. However, Pensieve repeats conservative and aggressive behaviors in bitrate selection. This helps the clients in achieving a certain level of fairness, but the performance is degraded due to the high smoothness penalty and the high re-buffering penalty. To obtain a sufficient buffer, the proposed scheme determines the low video bitrate at the beginning of the streaming session and gradually increases the bitrate. Furthermore, the proposed scheme maintains the high video bitrate, improving the QoE fairness without degrading the individual QoE.

## 5. Conclusions

In this paper, we presented the adaptive streaming scheme based on reinforcement learning in edge computing environments. The proposed scheme aims to optimize the performance of multi-client adaptive streaming. The optimization goal is achieved by training the neural network model using a state-of-the-art reinforcement learning algorithm and managing multiple clients under the supervision of a mobile edge. Through the experimental results, we have confirmed that the proposed scheme generates the optimal adaptation policy for multiple clients. As for future work, we plan to apply the proposed scheme to real network environments.

## Figures and Tables

**Table 1:** Parameter description for the multi-agent training.

Notation	Description	Value
m	Number of multiple inputs	8
γ	Discount factor	0.99
α, $\alpha^{\prime }$	Learning rates for the actor network and critic network	0.0001, 0.001
β	Entropy weight	5.0 to 0.1 (over 100,000 iterations)
δ, ρ	Weight parameters used in the QoE of clients	1.0, 4.3
N	Maximum number of clients	20

**Table 2:** Summary for the overall QoE and the individual QoE components.

Schemes	Overall QoE	Bitrate Utility	Smoothness Penalty	Re-Buffering Penalty
ECAA	1.01	1.80	0.50	0.29
Pensieve	1.68	2.01	0.14	0.19
Proposed	1.83	1.98	0.13	0.02

## Data Availability

Not applicable.
